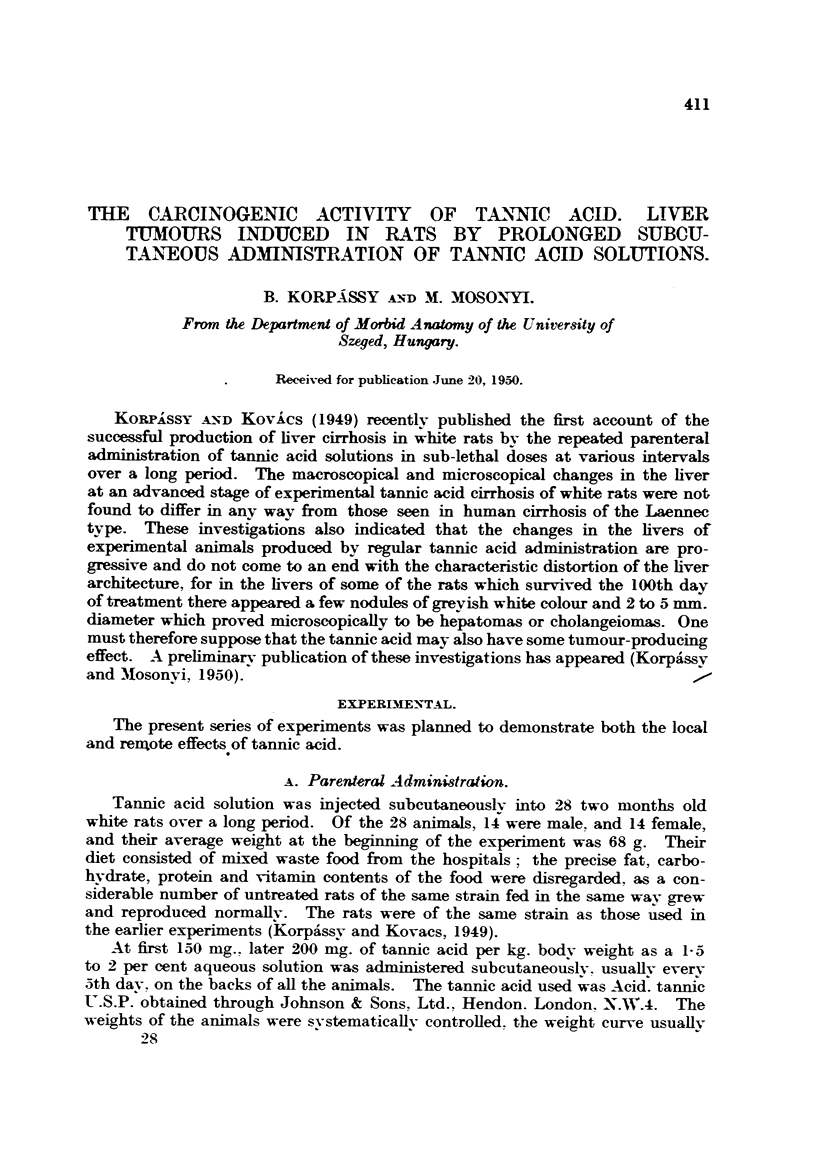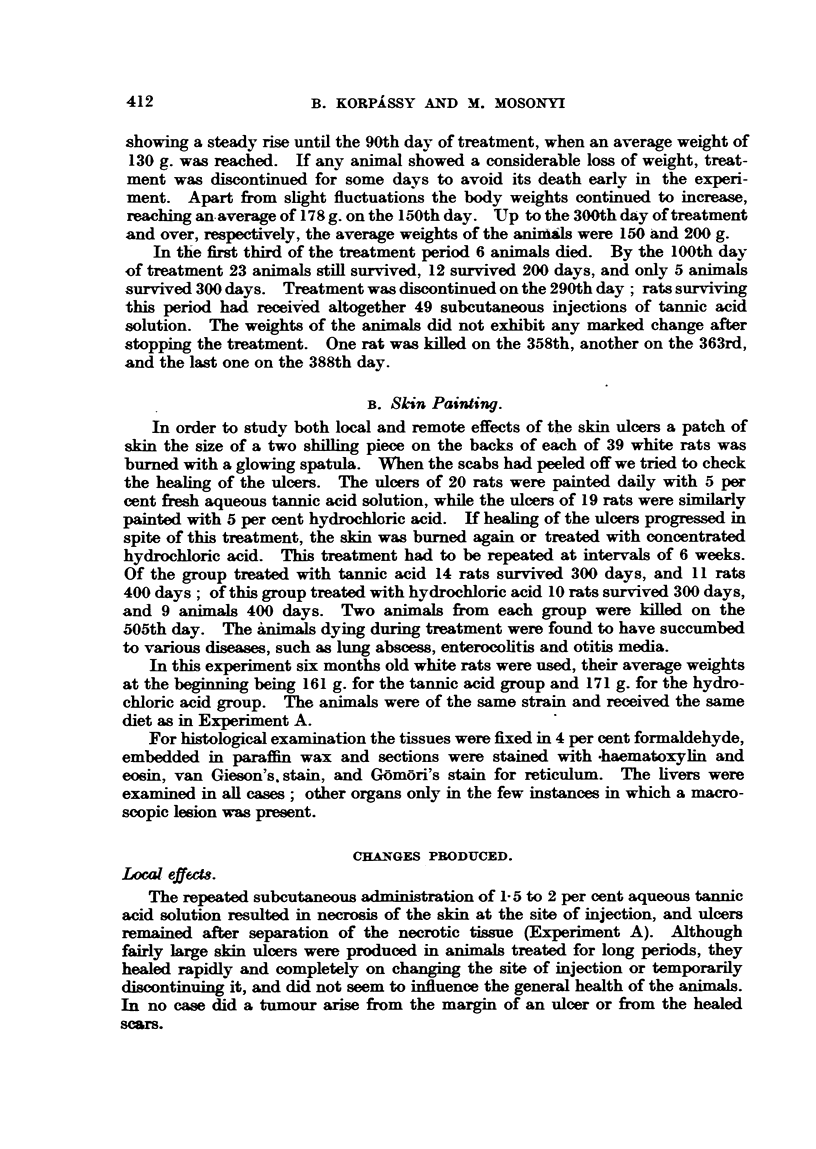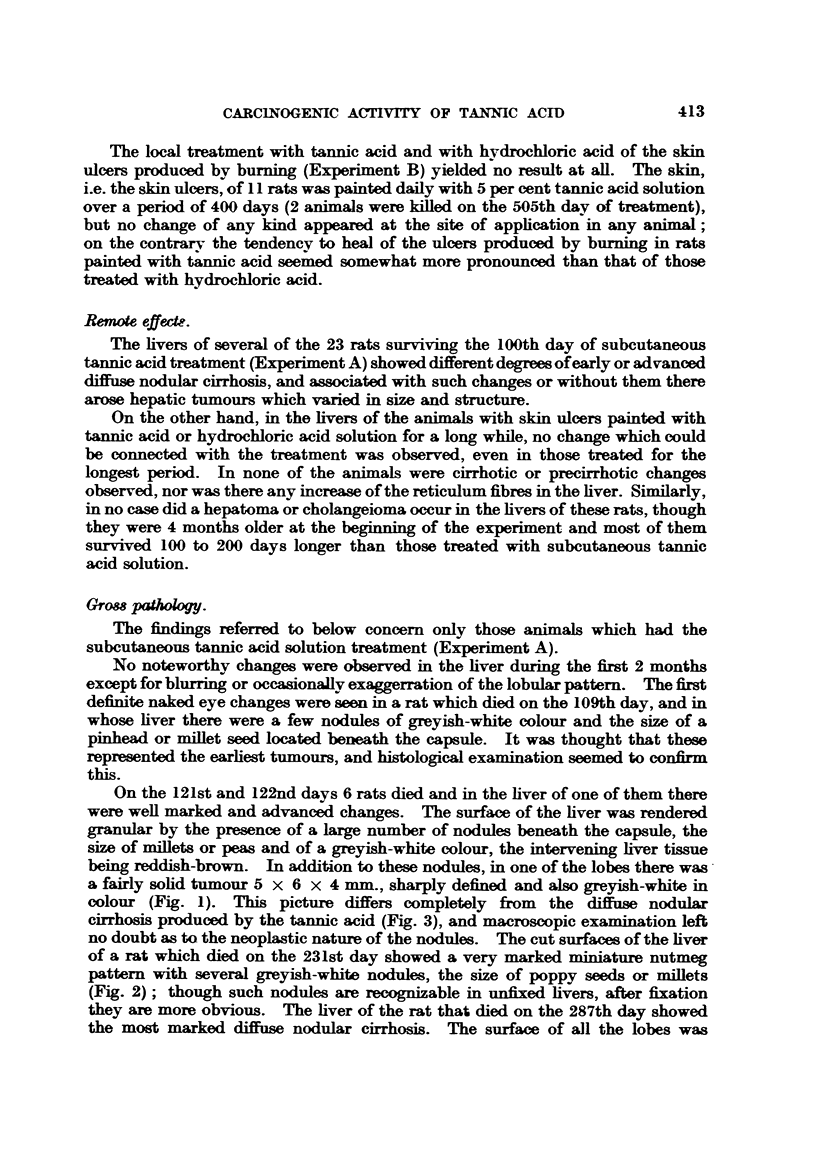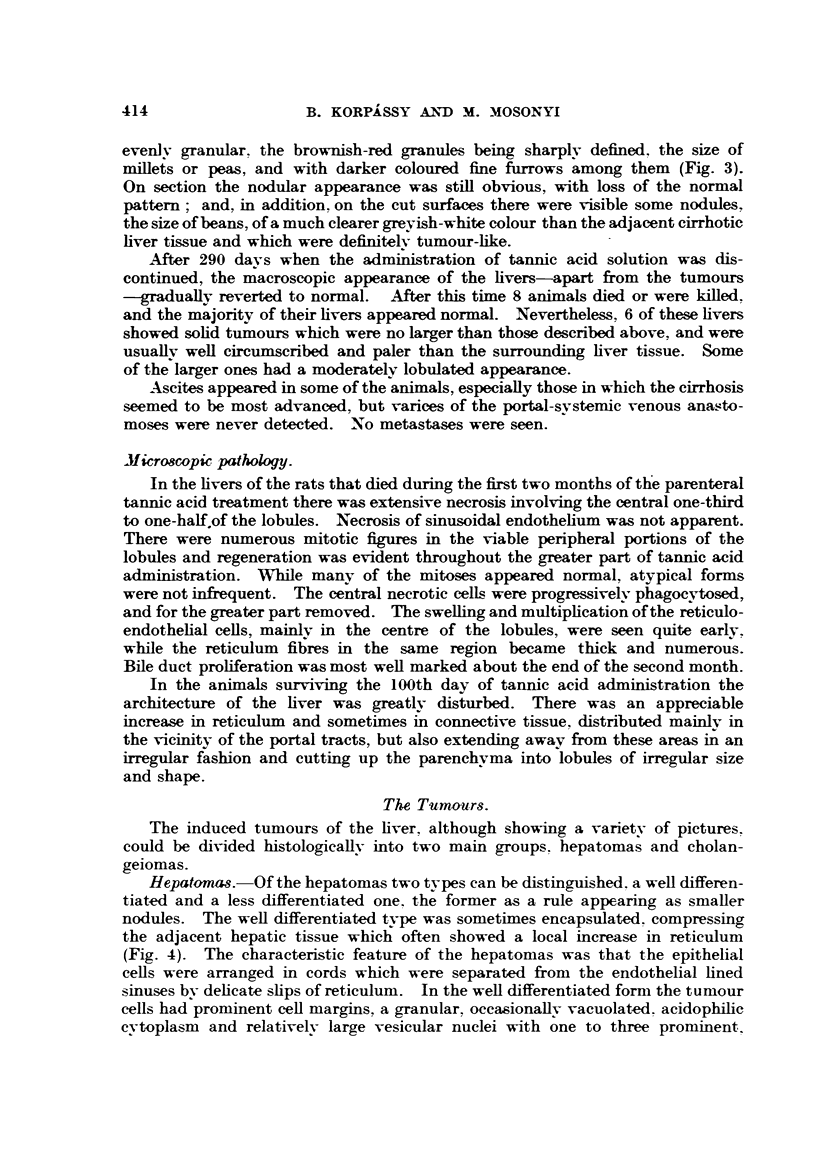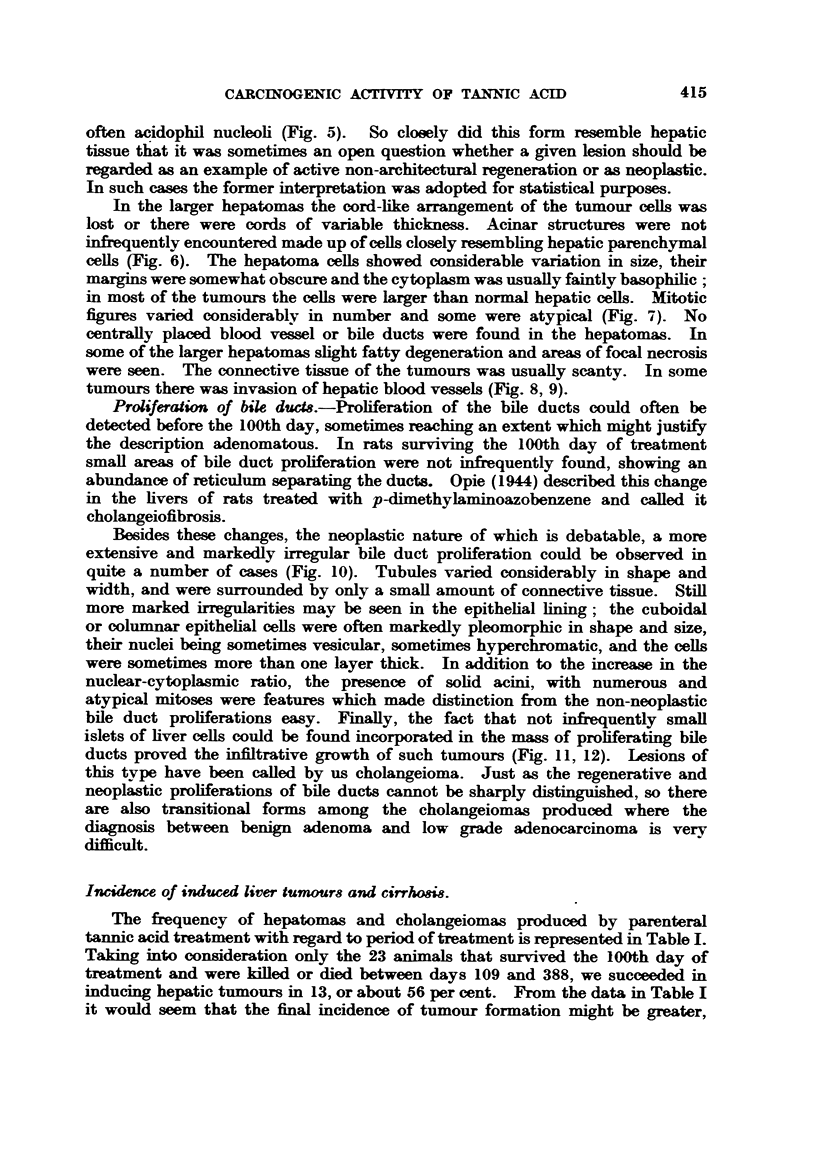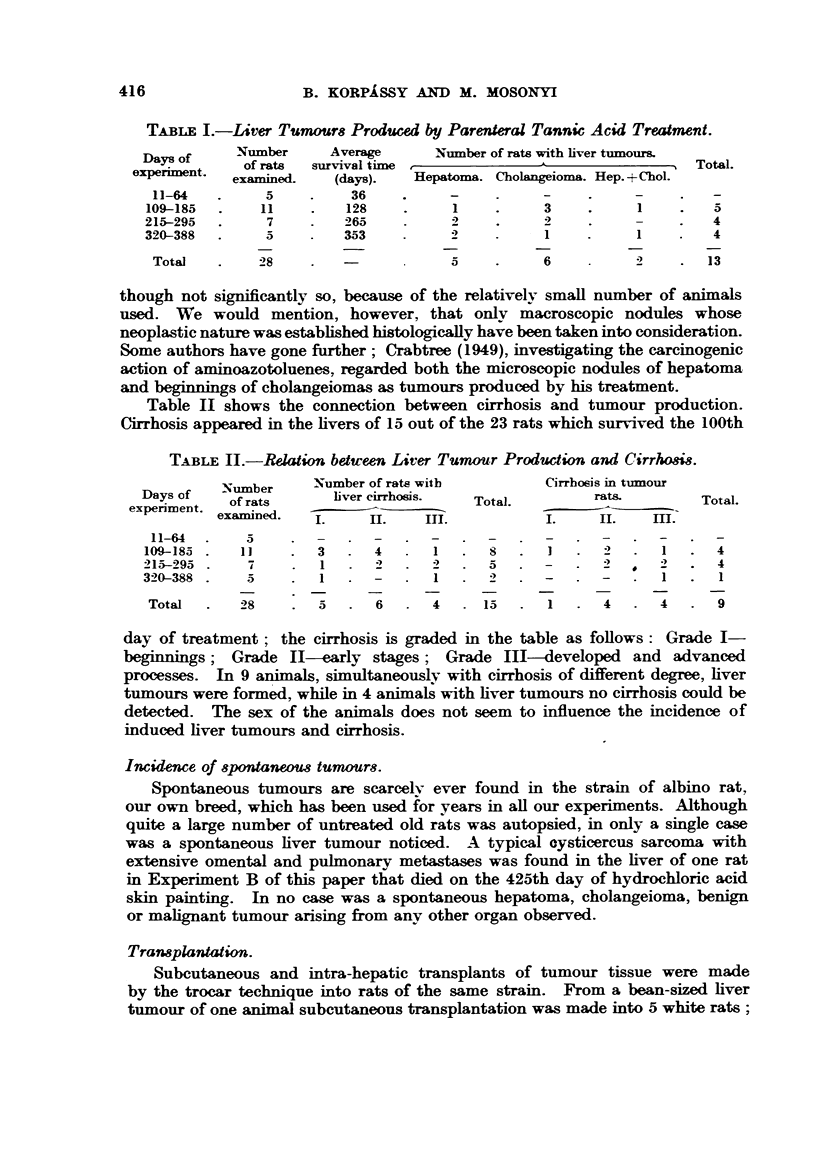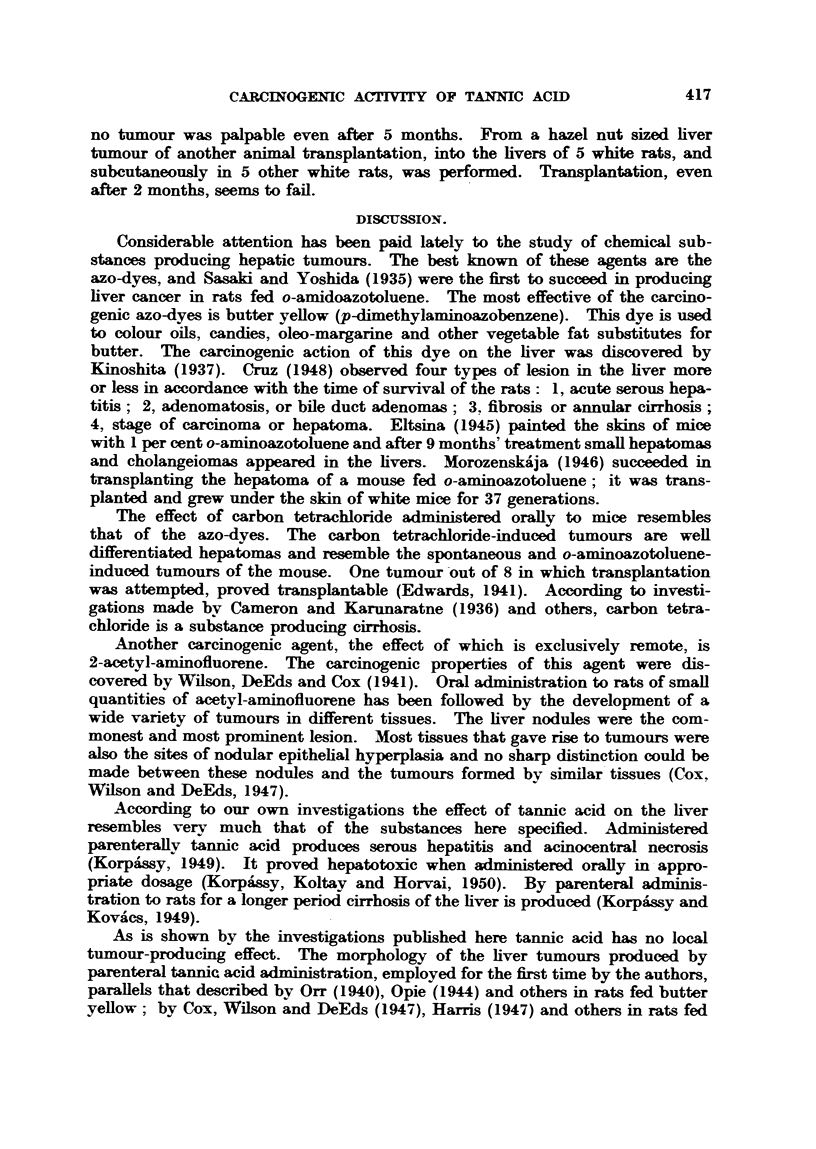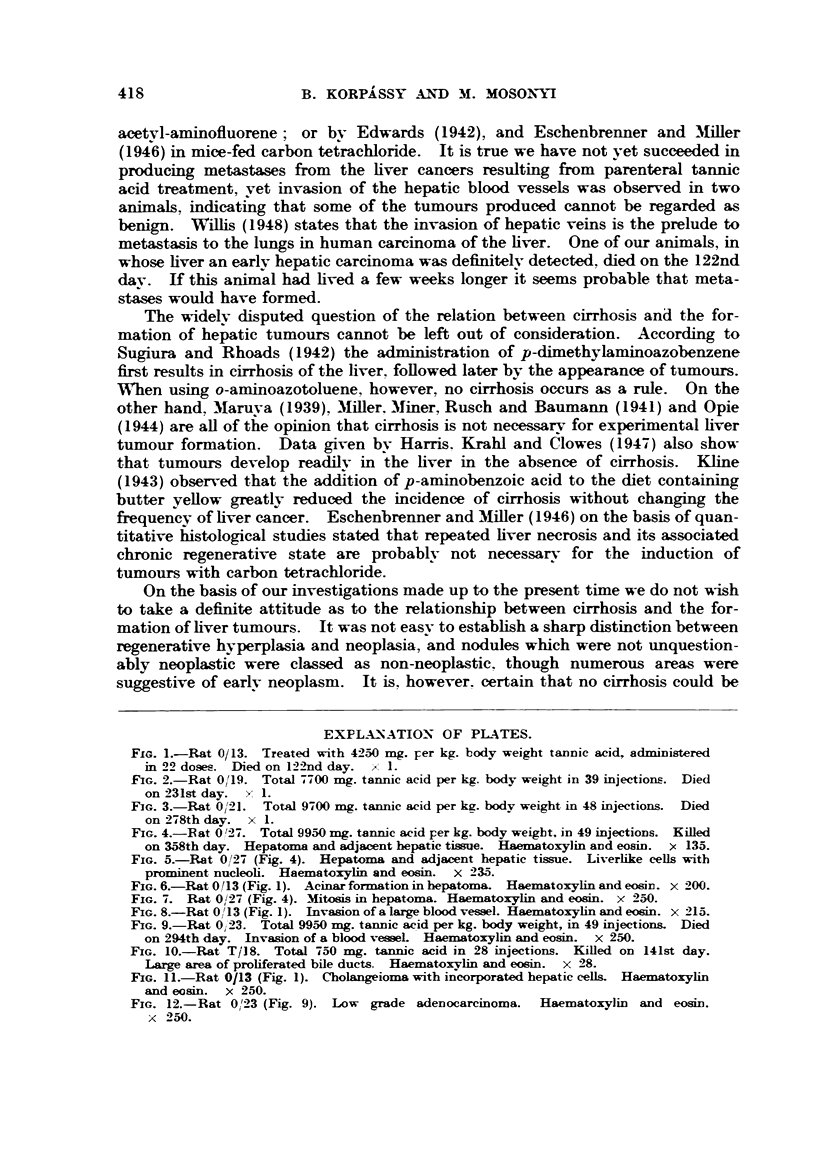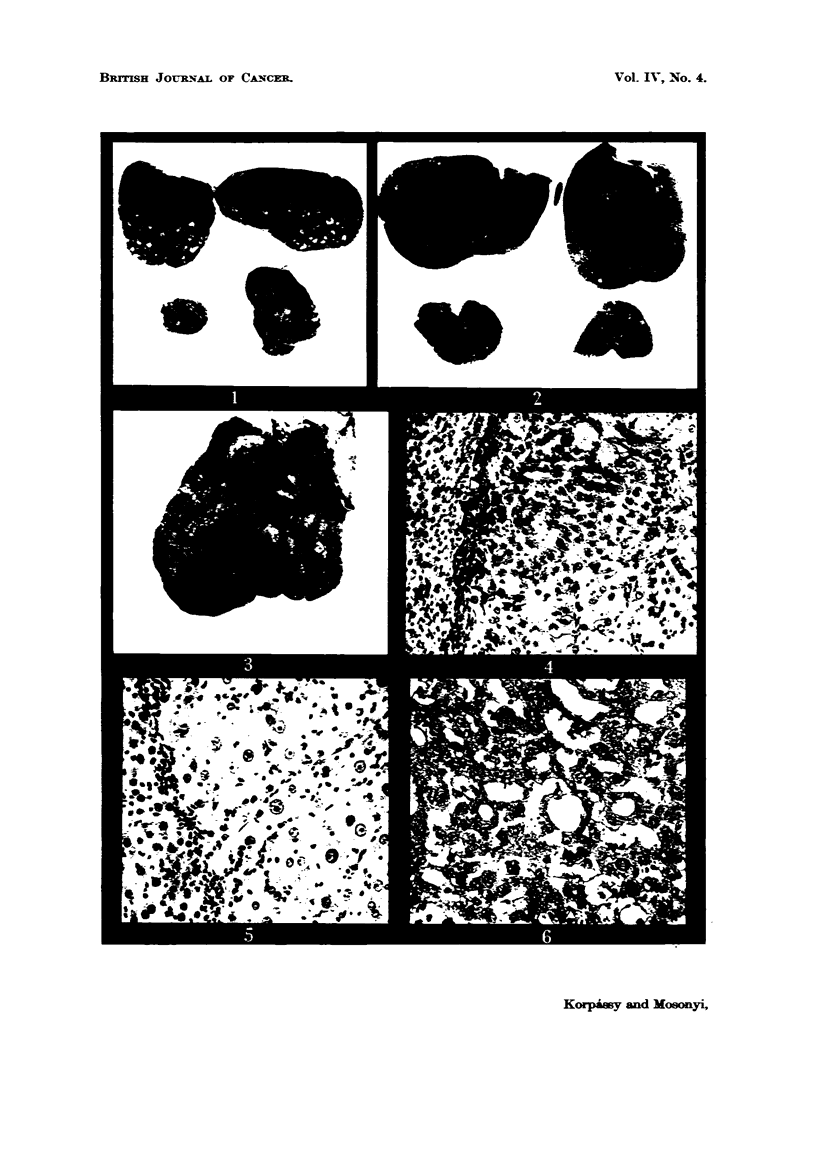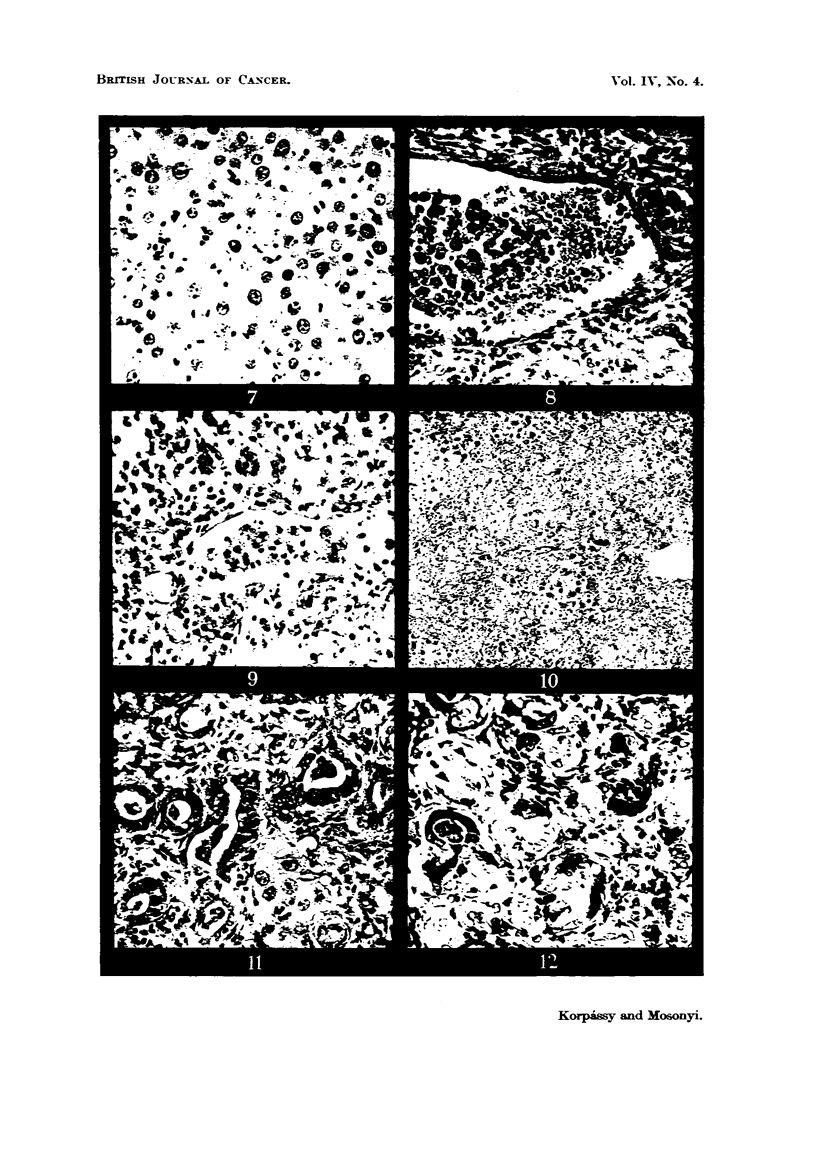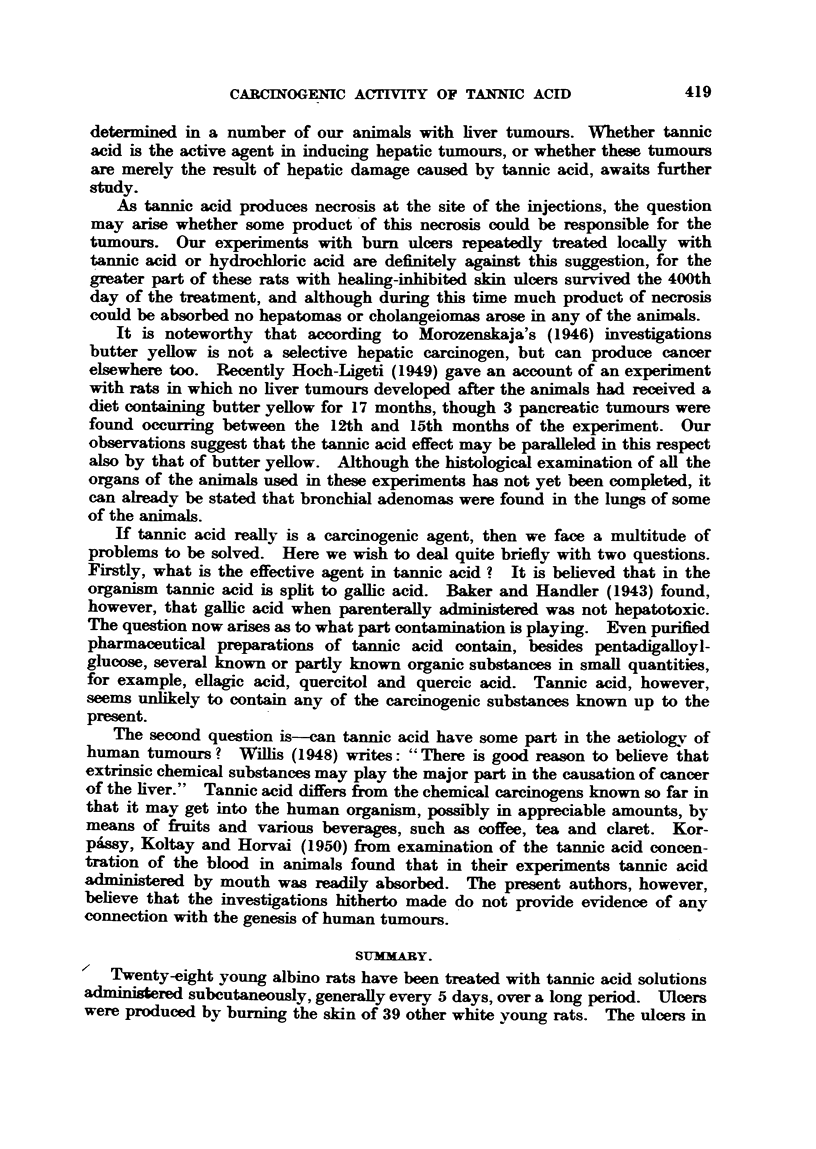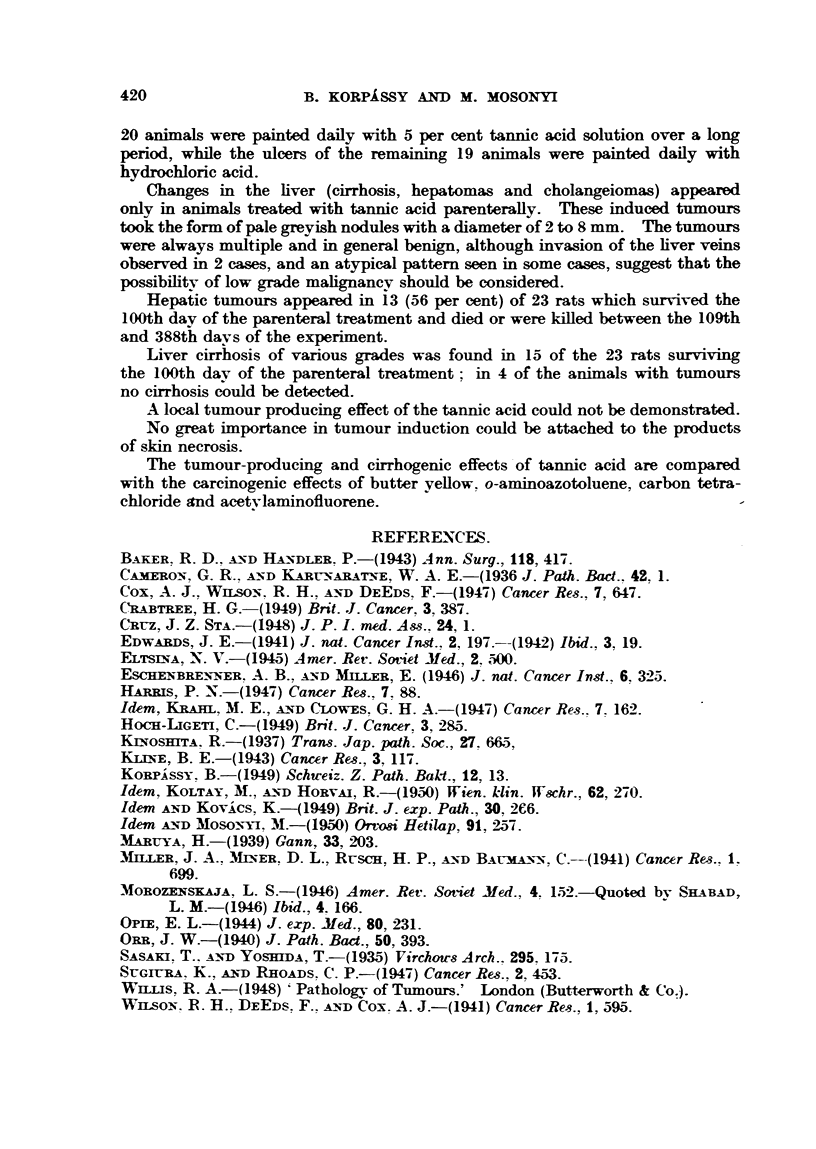# The Carcinogenic Activity of Tannic Acid. Liver Tumours Induced in Rats by Prolonged Subcutaneous Administration of Tannic Acid Solutions

**DOI:** 10.1038/bjc.1950.40

**Published:** 1950-12

**Authors:** B. Korpássy, M. Mosonyi

## Abstract

**Images:**


### THE CARCINOGENIC ACTIVITY OF TANNIC ACID. LIVER TUMOURS INDUCED IN RATS BY PROLONGED SUBCUTANEOUS ADMINISTRATION OF TANNIC ACID SOLUTIONS.

B. KORPÁSSY AND M. MOSONYI.

*From the Department of Morbid Anatomy of the University of Szeged, Hungary.*

Received for publication June 20, 1950.

KORPÁSSY AND KOVÁCS (1949) recently published the first account of the successful production of liver cirrhosis in white rats by the repeated parenteral administration of tannic acid solutions in sub-lethal doses at various intervals over a long period. The macroscopical and microscopical changes in the liver at an advanced stage of experimental tannic acid cirrhosis of white rats were not found to differ in any way from those seen in human cirrhosis of the Laennec type. These investigations also indicated that the changes in the livers of experimental animals produced by regular tannic acid administration are progressive and do not come to an end with the characteristic distortion of the liver architecture, for in the livers of some of the rats which survived the 100th day of treatment there appeared a few nodules of greyish white colour and 2 to 5 mm. diameter which proved microscopically to be hepatomas or cholangeiomas. One must therefore suppose that the tannic acid may also have some tumour-producing effect. A preliminary publication of these investigations has appeared (Korpássy and Mosonyi, 1950).

### EXPERIMENTAL.

The present series of experiments was planned to demonstrate both the local and remote effects of tannic acid.

### A. *Parenteral Administration.*

Tannic acid solution was injected subcutaneously into 28 two months old white rats over a long period. Of the 28 animals, 14 were male, and 14 female, and their average weight at the beginning of the experiment was 68 g. Their diet consisted of mixed waste food from the hospitals ; the precise fat, carbohydrate, protein and vitamin contents of the food were disregarded, as a considerable number of untreated rats of the same strain fed in the same way grew and reproduced normally. The rats were of the same strain as those used in the earlier experiments (Korpássy and Kovacs, 1949).

At first 150 mg., later 200 mg. of tannic acid per kg. body weight as a 1·5 to 2 per cent aqueous solution was administered subcutaneously, usually every 5th day, on the backs of all the animals. The tannic acid used was Acid. tannic U.S.P. obtained through Johnson & Sons, Ltd., Hendon, London, N.W.4. The weights of the animals were systematically controlled, the weight curve usually showing a steady rise until the 90th day of treatment, when an average weight of 130 g. was reached. If any animal showed a considerable loss of weight, treatment was discontinued for some days to avoid its death early in the experiment. Apart from slight fluctuations the body weights continued to increase, reaching an average of 178 g. on the 150th day. Up to the 300th day of treatment and over, respectively, the average weights of the animals were 150 and 200 g.

In the first third of the treatment period 6 animals died. By the 100th day of treatment 23 animals still survived, 12 survived 200 days, and only 5 animals survived 300 days. Treatment was discontinued on the 290th day ; rats surviving this period had received altogether 49 subcutaneous injections of tannic acid solution. The weights of the animals did not exhibit any marked change after stopping the treatment. One rat was killed on the 358th, another on the 363rd, and the last one on the 388th day.

### B. *Skin Painting.*

In order to study both local and remote effects of the skin ulcers a patch of skin the size of a two shilling piece on the backs of each of 39 white rats was burned with a glowing spatula. When the scabs had peeled off we tried to check the healing of the ulcers. The ulcers of 20 rats were painted daily with 5 per cent fresh aqueous tannic acid solution, while the ulcers of 19 rats were similarly painted with 5 per cent hydrochloric acid. If healing of the ulcers progressed in spite of this treatment, the skin was burned again or treated with concentrated hydrochloric acid. This treatment had to be repeated at intervals of 6 weeks. Of the group treated with tannic acid 14 rats survived 300 days, and 11 rats 400 days ; of this group treated with hydrochloric acid 10 rats survived 300 days, and 9 animals 400 days. Two animals from each group were killed on the 505th day. The animals dying during treatment were found to have succumbed to various diseases, such as lung abscess, enterocolitis and otitis media.

In this experiment six months old white rats were used, their average weights at the beginning being 161 g. for the tannic acid group and 171 g. for the hydrochloric acid group. The animals were of the same strain and received the same diet as in Experiment A.

For histological examination the tissues were fixed in 4 per cent formaldehyde, embedded in paraffin wax and sections were stained with haematoxylin and eosin, van Gieson's stain, and Gömöri's stain for reticulum. The livers were examined in all cases ; other organs only in the few instances in which a macroscopic lesion was present.

### CHANGES PRODUCED.

*Local effects.*

The repeated subcutaneous administration of 1·5 to 2 per cent aqueous tannic acid solution resulted in necrosis of the skin at the site of injection, and ulcers remained after separation of the necrotic tissue (Experiment A). Although fairly large skin ulcers were produced in animals treated for long periods, they healed rapidly and completely on changing the site of injection or temporarily discontinuing it, and did not seem to influence the general health of the animals. In no case did a tumour arise from the margin of an ulcer or from the healed scars.

The local treatment with tannic acid and with hydrochloric acid of the skin ulcers produced by burning (Experiment B) yielded no result at all. The skin, i.e. the skin ulcers, of 11 rats was painted daily with 5 per cent tannic acid solution over a period of 400 days (2 animals were killed on the 505th day of treatment), but no change of any kind appeared at the site of application in any animal; on the contrary the tendency to heal of the ulcers produced by burning in rats painted with tannic acid seemed somewhat more pronounced than that of those treated with hydrochloric acid.

*Remote effects.*

The livers of several of the 23 rats surviving the 100th day of subcutaneous tannic acid treatment (Experiment A) showed different degrees of early or advanced diffuse nodular cirrhosis, and associated with such changes or without them there arose hepatic tumours which varied in size and structure.

On the other hand, in the livers of the animals with skin ulcers painted with tannic acid or hydrochloric acid solution for a long while, no change which could be connected with the treatment was observed, even in those treated for the longest period. In none of the animals were cirrhotic or precirrhotic changes observed, nor was there any increase of the reticulum fibres in the liver. Similarly, in no case did a hepatoma or cholangeioma occur in the livers of these rats, though they were 4 months older at the beginning of the experiment and most of them survived 100 to 200 days longer than those treated with subcutaneous tannic acid solution.

*Gross pathology.*

The findings referred to below concern only those animals which had the subcutaneous tannic acid solution treatment (Experiment A).

No noteworthy changes were observed in the liver during the first 2 months except for blurring or occasionally exaggerration of the lobular pattern. The first definite naked eye changes were seen in a rat which died on the 109th day, and in whose liver there were a few nodules of greyish-white colour and the size of a pinhead or millet seed located beneath the capsule. It was thought that these represented the earliest tumours, and histological examination seemed to confirm this.

On the 121st and 122nd days 6 rats died and in the liver of one of them there were well marked and advanced changes. The surface of the liver was rendered granular by the presence of a large number of nodules beneath the capsule, the size of millets or peas and of a greyish-white colour, the intervening liver tissue being reddish-brown. In addition to these nodules, in one of the lobes there was a fairly solid tumour 5 × 6 × 4 mm., sharply defined and also greyish-white in colour (Fig. 1). This picture differs completely from the diffuse nodular cirrhosis produced by the tannic acid (Fig. 3), and macroscopic examination left no doubt as to the neoplastic nature of the nodules. The cut surfaces of the liver of a rat which died on the 231st day showed a very marked miniature nutmeg pattern with several greyish-white nodules, the size of poppy seeds or millets (Fig. 2); though such nodules are recognizable in unfixed livers, after fixation they are more obvious. The liver of the rat that died on the 287th day showed the most marked diffuse nodular cirrhosis. The surface of all the lobes was evenly granular, the brownish-red granules being sharply defined, the size of millets or peas, and with darker coloured fine furrows among them (Fig. 3). On section the nodular appearance was still obvious, with loss of the normal pattern ; and, in addition, on the cut surfaces there were visible some nodules, the size of beans, of a much clearer greyish-white colour than the adjacent cirrhotic liver tissue and which were definitely tumour-like.

After 290 days when the administration of tannic acid solution was discontinued, the macroscopic appearance of the livers—apart from the tumours—gradually reverted to normal. After this time 8 animals died or were killed, and the majority of their livers appeared normal. Nevertheless, 6 of these livers showed solid tumours which were no larger than those described above, and were usually well circumscribed and paler than the surrounding liver tissue. Some of the larger ones had a moderately lobulated appearance.

Ascites appeared in some of the animals, especially those in which the cirrhosis seemed to be most advanced, but varices of the portal-systemic venous anastomoses were never detected. No metastases were seen.

*Microscopic pathology.*

In the livers of the rats that died during the first two months of the parenteral tannic acid treatment there was extensive necrosis involving the central one-third to one-half of the lobules. Necrosis of sinusoidal endothelium was not apparent. There were numerous mitotic figures in the viable peripheral portions of the lobules and regeneration was evident throughout the greater part of tannic acid administration. While many of the mitoses appeared normal, atypical forms were not infrequent. The central necrotic cells were progressively phagocytosed, and for the greater part removed. The swelling and multiplication of the reticulo-endothelial cells, mainly in the centre of the lobules, were seen quite early, while the reticulum fibres in the same region became thick and numerous. Bile duct proliferation was most well marked about the end of the second month.

In the animals surviving the 100th day of tannic acid administration the architecture of the liver was greatly disturbed. There was an appreciable increase in reticulum and sometimes in connective tissue, distributed mainly in the vicinity of the portal tracts, but also extending away from these areas in an irregular fashion and cutting up the parenchyma into lobules of irregular size and shape.

*The Tumours.*

The induced tumours of the liver, although showing a variety of pictures, could be divided histologically into two main groups, hepatomas and cholangeiomas.

*Hepatomas.*—Of the hepatomas two types can be distinguished, a well differentiated and a less differentiated one, the former as a rule appearing as smaller nodules. The well differentiated type was sometimes encapsulated, compressing the adjacent hepatic tissue which often showed a local increase in reticulum (Fig. 4). The characteristic feature of the hepatomas was that the epithelial cells were arranged in cords which were separated from the endothelial lined sinuses by delicate slips of reticulum. In the well differentiated form the tumour cells had prominent cell margins, a granular, occasionally vacuolated, acidophilic cytoplasm and relatively large vesicular nuclei with one to three prominent, often acidophil nucleoli (Fig. 5). So closely did this form resemble hepatic tissue that it was sometimes an open question whether a given lesion should be regarded as an example of active non-architectural regeneration or as neoplastic. In such cases the former interpretation was adopted for statistical purposes.

In the larger hepatomas the cord-like arrangement of the tumour cells was lost or there were cords of variable thickness. Acinar structures were not infrequently encountered made up of cells closely resembling hepatic parenchymal cells (Fig. 6). The hepatoma cells showed considerable variation in size, their margins were somewhat obscure and the cytoplasm was usually faintly basophilic ; in most of the tumours the cells were larger than normal hepatic cells. Mitotic figures varied considerably in number and some were atypical (Fig. 7). No centrally placed blood vessel or bile ducts were found in the hepatomas. In some of the larger hepatomas slight fatty degeneration and areas of focal necrosis were seen. The connective tissue of the tumours was usually scanty. In some tumours there was invasion of hepatic blood vessels (Fig. 8, 9).

*Proliferation of bile ducts.*—Proliferation of the bile ducts could often be detected before the 100th day, sometimes reaching an extent which might justify the description adenomatous. In rats surviving the 100th day of treatment small areas of bile duct proliferation were not infrequently found, showing an abundance of reticulum separating the ducts. Opie (1944) described this change in the livers of rats treated with *p*-dimethylaminoazobenzene and called it cholangeiofibrosis.

Besides these changes, the neoplastic nature of which is debatable, a more extensive and markedly irregular bile duct proliferation could be observed in quite a number of cases (Fig. 10). Tubules varied considerably in shape and width, and were surrounded by only a small amount of connective tissue. Still more marked irregularities may be seen in the epithelial lining ; the cuboidal or columnar epithelial cells were often markedly pleomorphic in shape and size, their nuclei being sometimes vesicular, sometimes hyperchromatic, and the cells were sometimes more than one layer thick. In addition to the increase in the nuclear-cytoplasmic ratio, the presence of solid acini, with numerous and atypical mitoses were features which made distinction from the non-neoplastic bile duct proliferations easy. Finally, the fact that not infrequently small islets of liver cells could be found incorporated in the mass of proliferating bile ducts proved the infiltrative growth of such tumours (Fig. 11, 12). Lesions of this type have been called by us cholangeioma. Just as the regenerative and neoplastic proliferations of bile ducts cannot be sharply distinguished, so there are also transitional forms among the cholangeiomas produced where the diagnosis between benign adenoma and low grade adenocarcinoma is very difficult.

*Incidence of induced liver tumours and cirrhosis.*

The frequency of hepatomas and cholangeiomas produced by parenteral tannic acid treatment with regard to period of treatment is represented in Table I. Taking into consideration only the 23 animals that survived the 100th day of treatment and were killed or died between days 109 and 388, we succeeded in inducing hepatic tumours in 13, or about 56 per cent. From the data in Table I it would seem that the final incidence of tumour formation might be greater,

TABLE I.—*Liver Tumours Produced by Parenteral Tannic Acid Treatment.*

| Days of experiment. | Number of rats examined. | Average survival time (days). | Number of rats with liver tumours. | | | Total. |
|---|---|---|---|---|---|---|
| | | | Hepatoma. | Cholangeioma. | Hep.+Chol. | |
| 11–64 | 5 | 36 | – | – | – | – |
| 109–185 | 11 | 128 | 1 | 3 | 1 | 5 |
| 215–295 | 7 | 265 | 2 | 2 | – | 4 |
| 320–388 | 5 | 353 | 2 | 1 | 1 | 4 |
| Total | 28 | — | 5 | 6 | 2 | 13 |

though not significantly so, because of the relatively small number of animals used. We would mention, however, that only macroscopic nodules whose neoplastic nature was established histologically have been taken into consideration. Some authors have gone further ; Crabtree (1949), investigating the carcinogenic action of aminoazotoluenes, regarded both the microscopic nodules of hepatoma and beginnings of cholangeiomas as tumours produced by his treatment.

Table II shows the connection between cirrhosis and tumour production. Cirrhosis appeared in the livers of 15 out of the 23 rats which survived the 100th

TABLE II.—*Relation between Liver Tumour Production and Cirrhosis.*

| Days of experiment. | Number of rats examined. | Number of rats with liver cirrhosis. | | | Total. | Cirrhosis in tumour rats. | | | Total. |
|---|---|---|---|---|---|---|---|---|---|
| | | I. | II. | III. | | I. | II. | III. | |
| 11–64 | 5 | – | – | – | – | – | – | – | – |
| 109–185 | 11 | 3 | 4 | 1 | 8 | 1 | 2 | 1 | 4 |
| 215–295 | 7 | 1 | 2 | 2 | 5 | – | 2 | 2 | 4 |
| 320–388 | 5 | 1 | – | 1 | 2 | – | – | 1 | 1 |
| Total | 28 | 5 | 6 | 4 | 15 | 1 | 4 | 4 | 9 |

day of treatment ; the cirrhosis is graded in the table as follows : Grade I—beginnings ; Grade II—early stages ; Grade III—developed and advanced processes. In 9 animals, simultaneously with cirrhosis of different degree, liver tumours were formed, while in 4 animals with liver tumours no cirrhosis could be detected. The sex of the animals does not seem to influence the incidence of induced liver tumours and cirrhosis.

*Incidence of spontaneous tumours.*

Spontaneous tumours are scarcely ever found in the strain of albino rat, our own breed, which has been used for years in all our experiments. Although quite a large number of untreated old rats was autopsied, in only a single case was a spontaneous liver tumour noticed. A typical cysticercus sarcoma with extensive omental and pulmonary metastases was found in the liver of one rat in Experiment B of this paper that died on the 425th day of hydrochloric acid skin painting. In no case was a spontaneous hepatoma, cholangeioma, benign or malignant tumour arising from any other organ observed.

*Transplantation.*

Subcutaneous and intra-hepatic transplants of tumour tissue were made by the trocar technique into rats of the same strain. From a bean-sized liver tumour of one animal subcutaneous transplantation was made into 5 white rats ; no tumour was palpable even after 5 months. From a hazel nut sized liver tumour of another animal transplantation, into the livers of 5 white rats, and subcutaneously in 5 other white rats, was performed. Transplantation, even after 2 months, seems to fail.

### DISCUSSION.

Considerable attention has been paid lately to the study of chemical substances producing hepatic tumours. The best known of these agents are the azo-dyes, and Sasaki and Yoshida (1935) were the first to succeed in producing liver cancer in rats fed *o*-amidoazotoluene. The most effective of the carcinogenic azo-dyes is butter yellow (*p*-dimethylaminoazobenzene). This dye is used to colour oils, candies, oleo-margarine and other vegetable fat substitutes for butter. The carcinogenic action of this dye on the liver was discovered by Kinoshita (1937). Cruz (1948) observed four types of lesion in the liver more or less in accordance with the time of survival of the rats : 1, acute serous hepatitis ; 2, adenomatosis, or bile duct adenomas ; 3, fibrosis or annular cirrhosis ; 4, stage of carcinoma or hepatoma. Eltsina (1945) painted the skins of mice with 1 per cent *o*-aminoazotoluene and after 9 months' treatment small hepatomas and cholangeiomas appeared in the livers. Morozenskája (1946) succeeded in transplanting the hepatoma of a mouse fed *o*-aminoazotoluene ; it was transplanted and grew under the skin of white mice for 37 generations.

The effect of carbon tetrachloride administered orally to mice resembles that of the azo-dyes. The carbon tetrachloride-induced tumours are well differentiated hepatomas and resemble the spontaneous and *o*-aminoazotoluene-induced tumours of the mouse. One tumour out of 8 in which transplantation was attempted, proved transplantable (Edwards, 1941). According to investigations made by Cameron and Karunaratne (1936) and others, carbon tetrachloride is a substance producing cirrhosis.

Another carcinogenic agent, the effect of which is exclusively remote, is 2-acetyl-aminofluorene. The carcinogenic properties of this agent were discovered by Wilson, DeEds and Cox (1941). Oral administration to rats of small quantities of acetyl-aminofluorene has been followed by the development of a wide variety of tumours in different tissues. The liver nodules were the commonest and most prominent lesion. Most tissues that gave rise to tumours were also the sites of nodular epithelial hyperplasia and no sharp distinction could be made between these nodules and the tumours formed by similar tissues (Cox, Wilson and DeEds, 1947).

According to our own investigations the effect of tannic acid on the liver resembles very much that of the substances here specified. Administered parenterally tannic acid produces serous hepatitis and acinocentral necrosis (Korpássy, 1949). It proved hepatotoxic when administered orally in appropriate dosage (Korpássy, Koltay and Horvai, 1950). By parenteral administration to rats for a longer period cirrhosis of the liver is produced (Korpássy and Kovács, 1949).

As is shown by the investigations published here tannic acid has no local tumour-producing effect. The morphology of the liver tumours produced by parenteral tannic acid administration, employed for the first time by the authors, parallels that described by Orr (1940), Opie (1944) and others in rats fed butter yellow ; by Cox, Wilson and DeEds (1947), Harris (1947) and others in rats fed acetyl-aminofluorene ; or by Edwards (1942), and Eschenbrenner and Miller (1946) in mice-fed carbon tetrachloride. It is true we have not yet succeeded in producing metastases from the liver cancers resulting from parenteral tannic acid treatment, yet invasion of the hepatic blood vessels was observed in two animals, indicating that some of the tumours produced cannot be regarded as benign. Willis (1948) states that the invasion of hepatic veins is the prelude to metastasis to the lungs in human carcinoma of the liver. One of our animals, in whose liver an early hepatic carcinoma was definitely detected, died on the 122nd day. If this animal had lived a few weeks longer it seems probable that metastases would have formed.

The widely disputed question of the relation between cirrhosis and the formation of hepatic tumours cannot be left out of consideration. According to Sugiura and Rhoads (1942) the administration of *p*-dimethylaminoazobenzene first results in cirrhosis of the liver, followed later by the appearance of tumours. When using *o*-aminoazotoluene, however, no cirrhosis occurs as a rule. On the other hand, Maruya (1939), Miller, Miner, Rusch and Baumann (1941) and Opie (1944) are all of the opinion that cirrhosis is not necessary for experimental liver tumour formation. Data given by Harris, Krahl and Clowes (1947) also show that tumours develop readily in the liver in the absence of cirrhosis. Kline (1943) observed that the addition of *p*-aminobenzoic acid to the diet containing butter yellow greatly reduced the incidence of cirrhosis without changing the frequency of liver cancer. Eschenbrenner and Miller (1946) on the basis of quantitative histological studies stated that repeated liver necrosis and its associated chronic regenerative state are probably not necessary for the induction of tumours with carbon tetrachloride.

On the basis of our investigations made up to the present time we do not wish to take a definite attitude as to the relationship between cirrhosis and the formation of liver tumours. It was not easy to establish a sharp distinction between regenerative hyperplasia and neoplasia, and nodules which were not unquestionably neoplastic were classed as non-neoplastic, though numerous areas were suggestive of early neoplasm. It is, however, certain that no cirrhosis could be

---

EXPLANATION OF PLATES.

FIG. 1.—Rat 0/13. Treated with 4250 mg. per kg. body weight tannic acid, administered in 22 doses. Died on 122nd day. × 1.

FIG. 2.—Rat 0/19. Total 7700 mg. tannic acid per kg. body weight in 39 injections. Died on 231st day. × 1.

FIG. 3.—Rat 0/21. Total 9700 mg. tannic acid per kg. body weight in 48 injections. Died on 278th day. × 1.

FIG. 4.—Rat 0/27. Total 9950 mg. tannic acid per kg. body weight, in 49 injections. Killed on 358th day. Hepatoma and adjacent hepatic tissue. Haematoxylin and eosin. × 135.

FIG. 5.—Rat 0/27 (Fig. 4). Hepatoma and adjacent hepatic tissue. Liverlike cells with prominent nucleoli. Haematoxylin and eosin. × 235.

FIG. 6.—Rat 0/13 (Fig. 1). Acinar formation in hepatoma. Haematoxylin and eosin. × 200.

FIG. 7. Rat 0/27 (Fig. 4). Mitosis in hepatoma. Haematoxylin and eosin. × 250.

FIG. 8.—Rat 0/13 (Fig. 1). Invasion of a large blood vessel. Haematoxylin and eosin. × 215.

FIG. 9.—Rat 0/23. Total 9950 mg. tannic acid per kg. body weight, in 49 injections. Died on 294th day. Invasion of a blood vessel. Haematoxylin and eosin. × 250.

FIG. 10.—Rat T/18. Total 750 mg. tannic acid in 28 injections. Killed on 141st day. Large area of proliferated bile ducts. Haematoxylin and eosin. × 28.

FIG. 11.—Rat 0/13 (Fig. 1). Cholangeioma with incorporated hepatic cells. Haematoxylin and eosin. × 250.

FIG. 12.—Rat 0/23 (Fig. 9). Low grade adenocarcinoma. Haematoxylin and eosin. × 250.

Korpássy and Mosonyi,

Korpássy and Mosonyi.

determined in a number of our animals with liver tumours. Whether tannic acid is the active agent in inducing hepatic tumours, or whether these tumours are merely the result of hepatic damage caused by tannic acid, awaits further study.

As tannic acid produces necrosis at the site of the injections, the question may arise whether some product of this necrosis could be responsible for the tumours. Our experiments with burn ulcers repeatedly treated locally with tannic acid or hydrochloric acid are definitely against this suggestion, for the greater part of these rats with healing-inhibited skin ulcers survived the 400th day of the treatment, and although during this time much product of necrosis could be absorbed no hepatomas or cholangeiomas arose in any of the animals.

It is noteworthy that according to Morozenskaja's (1946) investigations butter yellow is not a selective hepatic carcinogen, but can produce cancer elsewhere too. Recently Hoch-Ligeti (1949) gave an account of an experiment with rats in which no liver tumours developed after the animals had received a diet containing butter yellow for 17 months, though 3 pancreatic tumours were found occurring between the 12th and 15th months of the experiment. Our observations suggest that the tannic acid effect may be paralleled in this respect also by that of butter yellow. Although the histological examination of all the organs of the animals used in these experiments has not yet been completed, it can already be stated that bronchial adenomas were found in the lungs of some of the animals.

If tannic acid really is a carcinogenic agent, then we face a multitude of problems to be solved. Here we wish to deal quite briefly with two questions. Firstly, what is the effective agent in tannic acid ? It is believed that in the organism tannic acid is split to gallic acid. Baker and Handler (1943) found, however, that gallic acid when parenterally administered was not hepatotoxic. The question now arises as to what part contamination is playing. Even purified pharmaceutical preparations of tannic acid contain, besides pentadigalloyl-glucose, several known or partly known organic substances in small quantities, for example, ellagic acid, quercitol and quercic acid. Tannic acid, however, seems unlikely to contain any of the carcinogenic substances known up to the present.

The second question is—can tannic acid have some part in the aetiology of human tumours ? Willis (1948) writes: "There is good reason to believe that extrinsic chemical substances may play the major part in the causation of cancer of the liver." Tannic acid differs from the chemical carcinogens known so far in that it may get into the human organism, possibly in appreciable amounts, by means of fruits and various beverages, such as coffee, tea and claret. Korpássy, Koltay and Horvai (1950) from examination of the tannic acid concentration of the blood in animals found that in their experiments tannic acid administered by mouth was readily absorbed. The present authors, however, believe that the investigations hitherto made do not provide evidence of any connection with the genesis of human tumours.

### SUMMARY.

Twenty-eight young albino rats have been treated with tannic acid solutions administered subcutaneously, generally every 5 days, over a long period. Ulcers were produced by burning the skin of 39 other white young rats. The ulcers in

20 animals were painted daily with 5 per cent tannic acid solution over a long period, while the ulcers of the remaining 19 animals were painted daily with hydrochloric acid.

Changes in the liver (cirrhosis, hepatomas and cholangeiomas) appeared only in animals treated with tannic acid parenterally. These induced tumours took the form of pale greyish nodules with a diameter of 2 to 8 mm. The tumours were always multiple and in general benign, although invasion of the liver veins observed in 2 cases, and an atypical pattern seen in some cases, suggest that the possibility of low grade malignancy should be considered.

Hepatic tumours appeared in 13 (56 per cent) of 23 rats which survived the 100th day of the parenteral treatment and died or were killed between the 109th and 388th days of the experiment.

Liver cirrhosis of various grades was found in 15 of the 23 rats surviving the 100th day of the parenteral treatment ; in 4 of the animals with tumours no cirrhosis could be detected.

A local tumour producing effect of the tannic acid could not be demonstrated.

No great importance in tumour induction could be attached to the products of skin necrosis.

The tumour-producing and cirrhogenic effects of tannic acid are compared with the carcinogenic effects of butter yellow, *o*-aminoazotoluene, carbon tetrachloride and acetylaminofluorene.

### REFERENCES.

BAKER, R. D., AND HANDLER, P.—(1943) *Ann. Surg.*, **118**, 417.

CAMERON, G. R., AND KARUNARATNE, W. A. E.—(1936 *J. Path. Bact.*, **42**, 1.

COX, A. J., WILSON, R. H., AND DEEDS, F.—(1947) *Cancer Res.*, **7**, 647.

CRABTREE, H. G.—(1949) *Brit. J. Cancer*, **3**, 387.

CRUZ, J. Z. STA.—(1948) *J. P. I. med. Ass.*, **24**, 1.

EDWARDS, J. E.—(1941) *J. nat. Cancer Inst.*, **2**, 197.—(1942) *Ibid.*, **3**, 19.

ELTSINA, N. V.—(1945) *Amer. Rev. Soviet Med.*, **2**, 500.

ESCHENBRENNER, A. B., AND MILLER, E. (1946) *J. nat. Cancer Inst.*, **6**, 325.

HARRIS, P. N.—(1947) *Cancer Res.*, **7**, 88.

*Idem*, KRAHL, M. E., AND CLOWES, G. H. A.—(1947) *Cancer Res.*, **7**, 162.

HOCH-LIGETI, C.—(1949) *Brit. J. Cancer*, **3**, 285.

KINOSHITA, R.—(1937) *Trans. Jap. path. Soc.*, **27**, 665,

KLINE, B. E.—(1943) *Cancer Res.*, **3**, 117.

KORPÁSSY, B.—(1949) *Schweiz. Z. Path. Bakt.*, **12**, 13.

*Idem*, KOLTAY, M., AND HORVAI, R.—(1950) *Wien. klin. Wschr.*, **62**, 270.

*Idem* AND KOVÁCS, K.—(1949) *Brit. J. exp. Path.*, **30**, 266.

*Idem* AND MOSONYI, M.—(1950) *Orvosi Hetilap*, **91**, 257.

MARUYA, H.—(1939) *Gann*, **33**, 203.

MILLER, J. A., MINER, D. L., RUSCH, H. P., AND BAUMANN, C.—(1941) *Cancer Res.*, **1**, 699.

MOROZENSKAJA, L. S.—(1946) *Amer. Rev. Soviet Med.*, **4**, 152.—Quoted by SHABAD, L. M.—(1946) *Ibid.*, **4**, 166.

OPIE, E. L.—(1944) *J. exp. Med.*, **80**, 231.

ORR, J. W.—(1940) *J. Path. Bact.*, **50**, 393.

SASAKI, T., AND YOSHIDA, T.—(1935) *Virchows Arch.*, **295**, 175.

SUGIURA, K., AND RHOADS, C. P.—(1947) *Cancer Res.*, **2**, 453.

WILLIS, R. A.—(1948) ‘Pathology of Tumours.’ London (Butterworth & Co.).

WILSON, R. H., DEEDS, F., AND COX, A. J.—(1941) *Cancer Res.*, **1**, 595.